# Challenges During Review of COVID-19 Research Proposals: Experience of Faculty of Medicine, Ain Shams University Research Ethics Committee, Egypt

**DOI:** 10.3389/fmed.2021.715796

**Published:** 2021-11-02

**Authors:** Diaa Marzouk, Iman Sharawy, Isabelle Nakhla, Mostafa El Hodhod, Hoda Gadallah, Amr El-Shalakany, Reda Elwakil, Mohammed M. Moussa, Alaa Ismail, Fathy M. Tash

**Affiliations:** ^1^Faculty of Medicine, Ain Shams University, Cairo, Egypt; ^2^Misr International University, US Naval Medical Research Unit No.3 (NAMRU-3), Cairo, Egypt; ^3^Faculty of Medicine, October 6 University, Giza, Egypt

**Keywords:** research ethics committees, Faculty of Medicine Ain Shams University, COVID-19 research, ethical challenges, accelerated review

## Abstract

The COVID-19 pandemic resulted in an overwhelming increase in research studies submitted to research ethics committees (RECs) presenting many ethical challenges. This article aims to report the challenges encountered during review of COVID-19 research and the experience of the Faculty of Medicine, Ain Shams University Research Ethics Committee (FMASU REC). From April 10, 2020, until October 13, 2020, the FMASU REC reviewed 98 COVID-19 research protocols. This article addressed the question of how to face an overwhelming amount of research submitted to the REC while applying the required ethical principles. Ethical challenges included a new accelerated mode of review, online meetings, balance of risks vs. benefits, measures to mitigate risks, co-enrolment in different studies, protection of a vulnerable COVID-19 population, accelerated decisions, online research, how to handle informed consent during the pandemic, and justification of placebo arm.

## Introduction

The majority of the RECs in North African countries are registered with the Office for Human Research Protections and have Federal Wide Assurance (FWA) active numbers (1). Egypt has a National Ethics Committee, active institutional committees and the Egyptian Medical Research Regulation Law for the regulation of clinical trials and human research ethics issued December 2020; the Egyptian National Ethical Committee collaborates with the United Nations Educational, Scientific and Cultural Organization (2).

**Figure 1:**
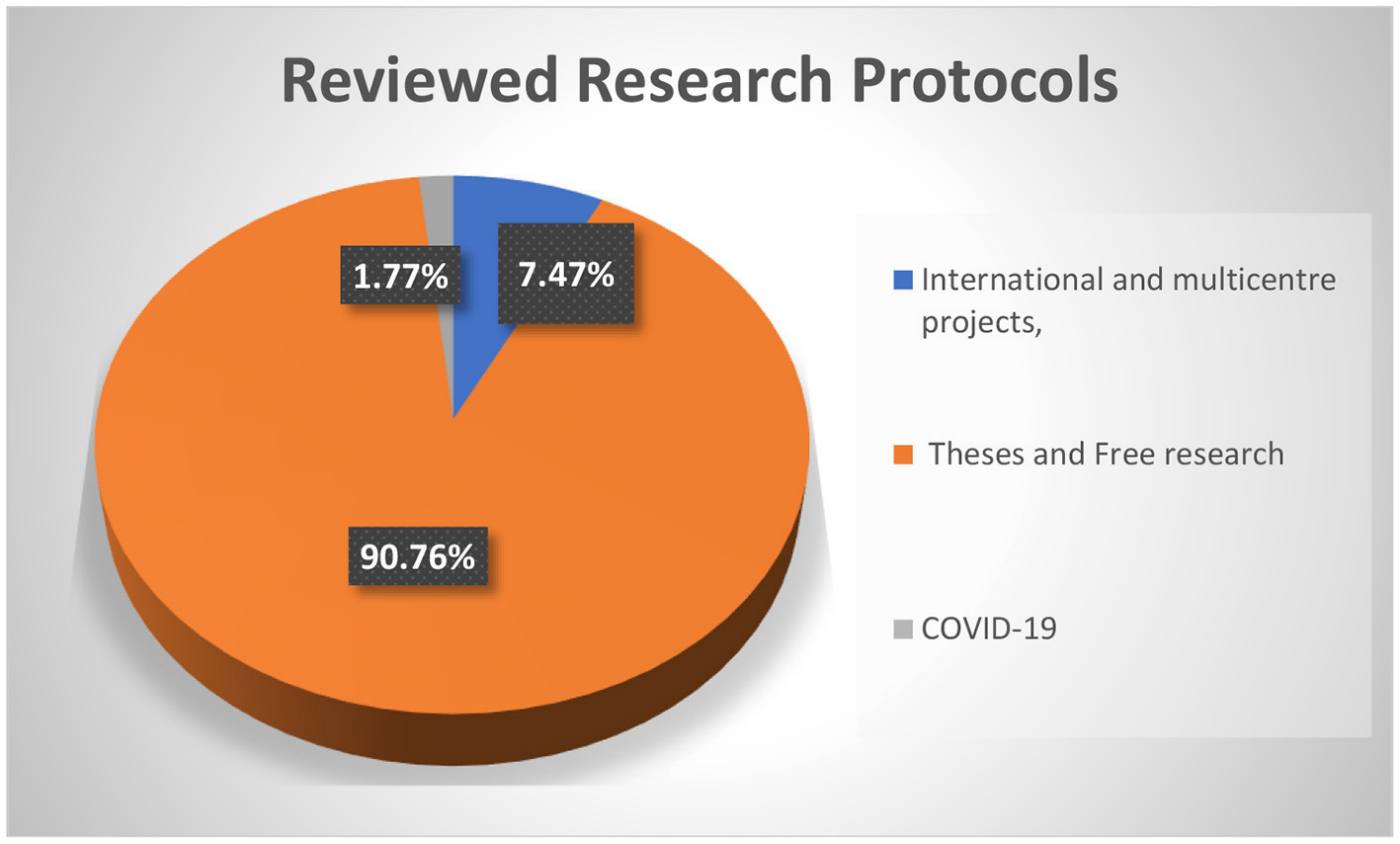
FMASU REC Workload from January 1st, 2008 to October 13th, 2020.

The FMASU REC was established in October 2007, to review research conducted at the Faculty of Medicine, Ain Shams University, in Cairo, Egypt. It holds a Federal Wide Assurance Number (FWA 00017585). The committee trained 220 staff members as reviewers working in 32 faculty departments over 23 training events. Since its establishment, the FMASU REC reviewed 414 international and multicentre projects, 5,033 theses, and free research. Since April 10, 2020, the FMASU REC reviewed 98 COVID-19 research studies, Figure 1.

During the COVID-19 pandemic in the year 2020, investigators began research to understand the novel virus, its epidemiology and pathogenicity, as well as to find ways for prevention and control, including discovering a treatment and/or vaccine. Worldwide, more than 4,900 studies and trials have been registered on “Clinicaltrials.gov” since the start of the pandemic (3). This rise in the number of emerging COVID-19 research projects has recently resulted in an overwhelming number of research project submissions to RECs.

To ensure ethical research during the COVID-19 pandemic, the World Health Organization (WHO) summarized the key universal ethical standards that should be adhered to by researchers, review bodies, funders, publishers, and manufacturers during a pandemic (4), identifying the main ethical standards as scientific validity, reasonable risk-benefit ratio, fair and voluntary participation, and independent review (5).

The European Network of Research Ethics Committees (EUREC) issued a statement that was adopted by the EUREC Board on April 27, 2020, stressing the fact that the administrative processes for reviewing research protocols during the COVID-19 pandemic must be accelerated and simplified if these protocols are related to the treatment, prevention or diagnosis of infections caused by SARS-CoV-2 (6). However, all research must be guided by the principle that RECs will not compromise the quality of the review process under these extraordinarily stressful circumstances. Furthermore, an accelerated procedure cannot be at the expense of the safety of research participants. The recognized ethical principles must always be respected, and the free and informed consent procedure must remain in accordance with international and national regulations.

In Egypt, there are 115 university hospitals (7) conducting research on human beings which needs approval from institutional RECs which are either internationally recognized and have an international FWA number and/or are registered with the Ministry of Health and Population (MOHP), or are newly developed and have been submitted for registry with the MOHP. International projects need other final approval from the MOHP REC.

The Clinical Medical Research Regulation Law (the Egyptian law for the conduct of research) was issued on December 23, 2020; its bylaws are currently being written. The Egyptian law is aligned with international guidelines for health research ethics review. The research studies protocols described in this manuscript were submitted during the period April 10 to October 13, 2020, to Ain Shams REC and were reviewed according to the international guidelines.

This article tried to answer the question of how the REC can effectively apply ethical principles when faced with an overwhelming number of research projects submitted.

### Aim

The aim of this article is to report the challenges encountered and the experience of the FMASU REC during review of COVID-19 research, starting from April 10 to October 13, 2020, and to give an overview about the challenges that the committee faced and how it overcame them.

### Modified Standard Operating Procedures

The article tried to illustrate how a governmental university REC in a low-middle income country modified its standard operating procedures (SOPs) to cope with a fast-track review of the overwhelming COVID-19 research and continue reviewing non-COVID-19 research. During the first wave of the pandemic there were no international guidelines for reviewing COVID-19 research. This challenge was increased by a scarcity of information about the disease, governmental lockdown periods, and lack of extra budget allocation.

The FMASU REC was confronted by multiple challenges in reviewing COVID-19 research during the pandemic, necessitating out-of-the box solutions to maintain an effective, accelerated review while at the same time practicing the ethical principles required.

The researchers, as well as the REC members, were encouraged to transform these challenges into opportunities. The SOPs followed by the REC were updated, regarding protocol submission changes to adopt digital submission route, rather than hard-copy paper submissions. Training of the employees on the use of digital technology for submissions and archiving followed. An electronic signature for the head of the committee was introduced. An accelerated, fast-track mode for reviewing protocols was adopted to cope with the pace of the pandemic. While the reviewers had previously been allowed up to 1 month for response for randomized controlled trials (RCTs), they were now expected to respond within one-seven days during the pandemic. The expedited process was meant to help in generating desperately needed knowledge out of the submitted research.

The REC resorted to on-line conferencing to overcome the inability to hold face-to-face meetings due to the Faculty lockdown. The first wave of research was received on April 10, 2020, with seven research projects reviewed in two online meetings over 3 days, including five clinical trials and two observational studies. That new mode of reviewing was dynamic, accelerated, and fast-tracked, in accordance with international guidelines and the REC's updated SOPs.

As the number of submitted research protocols increased rapidly, the REC had to increase the frequency of meetings to every other day, then every week or 2 weeks instead of the previous monthly schedule. Reviewing was done in a shorter time as the institution and investigators were expecting a rapid response. The usual review process that was adhered to by the REC was as follows: all the above minimal-risk protocols were initially reviewed by two reviewers and then discussed in full board meetings. To acquire a faster review track of the COVID-19-related proposals, clinical trials, or repurposed drugs, the number of initial reviewers was increased to three members, then discussed in full board online meetings. The number of members attending the virtual meetings ranged from 9 to 13 members, out of the 15 members comprising the committee.

## Methods

For this article we reviewed the list of all protocols submitted to FMASU REC for review during the COVID-19 time from April 10, 2020, until October 13, 2020. We listed the titles, principal investigators (PI) names, date of submission and date of response to the investigators. We analyzed the frequencies from these data. We also reviewed the meeting minutes to pin out the most interesting and challenging issues that were discussed during the meetings. Additionally, we reviewed the current procedures and changes that were instituted to the SOPs. Finally, we asked the members to provide their input about their concerns in the review process during the COVID-19 time. Last, we incorporated all the data into the article.

### Findings

#### Review Processes

Reviewing research during COVID-19 pandemic lockdown included shortened average duration of review, rapid request for clarifications and reply of investigators and quick provision of decisions. The duration of review was shortened to a minimum of 1 day and a maximum of 7 days. Before the pandemic, for more than minimal risk (low risk studies) and commercially-sponsored studies, the REC adopted a two-reviewer system, followed by discussion by the full Board. During the pandemic, to accomplish a shorter review time, this system was replaced by a three-member review, followed by the online meeting. As for minimal risk studies, the pre-pandemic system was also a two-reviewer, expedited review system, while during the pandemic for COVID-19 protocols, two reviewers were still assigned to review each protocol, but in a shorter reviewing period of 1-7 days.

To overcome the challenges of the short review time, the reviewing process of COVID-19 research stressed the rationale or justification as tackled by many researchers all over the world, the research question, hypothesis, social value, and benefits to the community. The novelty of the research idea was also an important point of discussion during the review process.

“Good science is itself an ethical requirement, as it is meaningless to apply ethical principles to a scientifically flawed product or plan. Bad science can only be bad ethics” (8). A rigorous revision of the research methodology was conducted, including study design, sample size and type, study procedures, randomization, and blinding. Many studies needed redesigning to specify inclusion and exclusion criteria of subjects, or to rigorously define the diagnosis of mild, moderate, and severe COVID-19 cases according to the National Guidelines reported and updated by the Egyptian MOHP.

Ensuring the well-being of researchers and research participants in the context of a pandemic was a very important objective of the REC. For research participants' well-being, hospital beds and equipment disinfection were under control of the Infection Control Unit, thus conforming to all international guidelines. As for mental health studies (some included healthcare professionals as well as patients), the committee recommended providing medical assistance for those who had high scores (e.g., recommending adding a paragraph in the questionnaire telling participants that if they had high scores for depression, to seek medical consultation). As for researchers, the REC insisted on following international, local governmental, and institutional recommendations. They were supplied with personal protective equipment including surgical, N95 masks, face shields, and goggles.

The REC was rigorous about sample size calculation in the protocol, urging the investigators to have a precise, predefined sample-size calculation, in order to receive REC approval of the study. As a standard procedure, the investigators were required, as a prerequisite before submission to the REC, to refer to the accredited statistics unit at the Department of community medicine in FMASU, to obtain the sample size calculation for any research. While some researchers chose to conduct a pilot study and a convenience sampling because of lack of sufficient data for new or repurposed drugs, the investigators had to provide strong justifications in such instances. The REC required an interim analysis and power calculation, to see if the sample size was large enough, or needed revision to find out if the efficacy of the tested drugs had been reached.

### Special Concerns During Review

The balance of risks and benefits is a pivotal element for the protection of human subjects in research. The REC exerted a great effort to mitigate research risks to provide maximum possible protection of research participants. The REC members spent long hours reviewing recent COVID 19-related publications, with special emphasis on adverse events reported involving drugs under trial and drug interactions. Clarifications were required on how to minimize the risks of these side effects, and suggestions were offered to the researchers. For instance, REC recommended additional investigations such as a baseline electrocardiogram, complete blood counts, requested exclusion of high-risk participants, or recommended increased frequency of monitoring visits.

The COVID-19 submitted protocols posted a novel risk-benefit evaluation to the reviewers. For example, in many instances, the REC could not ignore the risk to the researchers who had to interact face-to-face with COVID-19 patients in intensive care units (ICU). The REC had to ensure performance of enrolment and study procedures in unconventional circumstances, such as instances when researchers might not have been allowed in the research setting. The ICU physicians and nurses had to be trained to perform the study procedures. The PI had to keep close contact with the ICU staff and get monitoring reports about the enrolled participants. Whenever possible, the PI could see the patients following the standard safety infection control precautions.

One of the challenges faced by the FMASU REC was the lack of or insufficient animal studies and combining of Phases II and III for testing new drugs. The REC did not encounter Phase I protocols at that time. While the side effects and risks of the proposed therapies were not yet fully studied, Phase II usually includes more patients, and combining Phases II and III usually results in larger sample sizes than Phase II alone.

The REC responded by evaluating the risks and benefits, while maintaining strict requirements for risk minimization. The direct, potential patient benefit was the hope for effectiveness of new drugs, and the indirect benefit was withdrawing drugs from the list of potential drugs if proven non-effective.

### Workflow

The REC faced a big challenge with the large number of protocols, exceeding by far the routine work of the committee. Repurposed drugs, innovative drugs, and vaccines necessitate enormous steps to be approved. During the first 6 months of the pandemic, there was a relatively greater flow of RCTs, Phase III (2/5.85%) and Phase II (15/0.52%), compared to the period before the pandemic. The majority of studies were low risk observational studies (79.86%). Low risk studies are usually reviewed in an expedited manner by two reviewers, but in a shorter reviewing period of 1-7 days. The duration of the initial review was shortened to a minimum of 1 day and a maximum of 7 days compared to a minimum 1-month clinical trial review time, according to the REC SOPs. The total review time in the first rush of protocols during the pandemic was 1 week, but later the total review time was within 1 month, depending on how rapidly the investigators responded to the REC's comments.

The REC members devoted all their time to pandemic work, as most of the submitted protocols, even the non-commercial research, were to find an effective treatment for the emerging COVID-19 disease, either through using a repurposed drug, steroids, or antiviral drugs used earlier in management of Ebola virus, HCV, HIV, or antimalarial drugs. The flow of routine research as multicentre studies between Egyptian research centers, international centers and single center, non-commercial international project submissions, amendments, renewals and theses was slower than before the pandemic, but was reviewed in the same, accelerated manner.

The thesis topics submitted during the early wave of the COVID-19 pandemic were not yet related to the pandemic, but later in 2020 the topics were related to COVID-19.

The refusal rate was minimal; one study was deferred until more information could be obtained, as per the REC reviewers' request. Further details cannot be mentioned in this manuscript for protection of the confidentiality of the research topics.

The most frequent types of research submitted to the REC during the COVID-19 pandemic were observational studies (76.86%) to know the nature of this new emerging disease, followed by Phase III RCTs (5.85%), trying to find the most effective treatment through novel or repurposed drugs, such as drugs previously used in diseases other than COVID-19 such as EBOLA, Hepatitis C Virus, Human Immune Deficiency Virus and malaria Table 1.

**Table 1:** Frequency of clinical trial phases reviewed by FMASU REC from April to October 2020.

**Study Design**	**Number of protocols**	**Frequency of protocols**
**Randomized controlled trials**		
RCT Phase II	2	0.52%
RCT Phase III	15	5.85%
Total RCT	17	6.37%
**Observational studies**		
Cross-sectional study	52	54.10%
Case control study	5	5.85%
Cohort study	13	16.91%
Total observational studies	70	76.86%
**Miscellaneous**		
Exploratory	4	3.64%
Diagnostic	3	4.68%
Registry	2	3.90%
Systematic review	1	2.21%
Review article	1	2.34%
Total miscellaneous	11	16.77%
Total	98	100.00

The protocols might not have been written as state of the art, as investigators submitted them in an expedited manner. This necessitated more frequent and more rapid than usual communication between the FMASU REC and the investigators. In some instances, the REC required the investigators to provide more information to be able to make informed decisions. The direct communication between the REC members and the investigators was effective in enhancing the quality of the protocols and their scientific validity, in view of the scarce and controversial information concerning COVID-19.

Defining the target population of COVID-19 patients and the vulnerability of this population was another challenge. The investigators had to define their study population with regard to the severity of the disease, the state of consciousness, and addressing what the REC defined as a new vulnerability group; the COVID-19 patients were desperate for treatment and might have agreed to participate in any research project without proper consideration. The protection of moderate and severe cases of COVID-19, as vulnerable groups, was extremely important to the REC. Therefore, the REC insisted that mild and moderate cases give consent for themselves and did not allow a legally-authorized representative to give consent on their behalf.

Allowing enrolment of severe disease cases engendered a wide range of discussion. Although the severe cases were in great need of the benefit of any drug at a time when no proven cure was available, the opposing committee members were hesitant to allow severely ill cases to be enrolled in the studies, if the investigators could not provide enough preliminary evidence of a potential direct benefit. Some investigators requested that in severe cases, the ICU manager might sign the consent on behalf of the subjects, but the REC refused this idea and insisted that the patient be conscious enough to give his or her own consent. Otherwise, the investigators would have to obtain the legal guardian's consent outside the isolation hospital due to rules on who was allowed in an isolation hospital.

### Reviewing Telemedicine Studies

The review process had previously been accomplished through hard copies and online communication, as per the preference of the involved REC reviewers. During the pandemic, the shift to electronic communication became mandatory among researchers, the REC administrative office, the REC board, and the reviewers. The institution administration, as well, supported this shift and provided online meeting platforms in support of the digital transformation.

The COVID-19 pandemic resulted in greater use of online surveys and telemedicine. Telemedicine for clinical care started in 2016 at the Faculty of Medicine, Ain Shams University in the Neurology Department to help communication with patients being seen in clinical practice and was later extended to include other departments. To counteract the effects of the lockdown due to COVID-19, FMASU offers different telemedicine services, including consultation and outpatient clinics. Seven departments offer these services: Family Medicine, Clinical Oncology, Internal Medicine, Psychiatry, Paediatrics, Geriatrics and Obstetrics and Gynaecology. Services are offered through a secure link, where data, images and laboratory results can be uploaded and stored in the patient's medical record. The REC received this new type of telemedicine research as another challenge, Table 2. The use of online surveys to study the behavioral and psychiatric well-being of the community by different age groups, different study populations and in different places, was a new type of research for the REC to review, constituting 13% of the submitted COVID-19 research at that time and the second most frequent type of studies to be reviewed after therapeutic research (18%). The lack of experience in reviewing this type of research was challenging. Breeching of confidentiality and assurance were the major concerns. Additionally, telemedicine services are not common in developing countries like Egypt due to high illiteracy rate, 24.6% in July 2019 as announced by the Central Agency for Public Mobilization and Statistics (9). While the REC tried to ensure optimization of the research protocol and data collection and follow up, the REC experienced difficulties in interpretation of the PIs or physicians' instructions and data collection by phone. However, the REC also requested that the patient have an educated relative beside him to ensure proper comprehension of the instructions of the PI and for easier communication.

**Table 2:** Most common COVID-19 research topics reviewed by FMASU REC from April to October 2020.

**Topic**	**No**	**%**
Therapeutic	18	18.4
Online surveys	13	13.3
Repurposed drugs	12	12.2
Diagnostic	5	5.1
Vaccine trials	1	1
Genomics	1	1
Others	48	49
Total reviewed studies	98	100

## Discussion

Ain Shams University includes eight hospitals and several health centers. The total number of beds affiliated with the university is 2,300. Research is allowed in all hospitals and health centers except the specialized hospitals (the Specialized Hospital on within the FMASU campus and the Obour Specialized Hospital in Obour city).

During the first wave of the pandemic from February to October 2020, two main hospitals were transformed into isolation hospitals for moderate and severe adult cases, Obour Specialized Hospital, and the Geriatric Hospital; one new hospital was established, Al Maidany Hospital. Ain Shams student dorms were transformed into hospitals to receive moderate COVID-19 cases. The Internal Medicine and Surgery Departments were allocated for adult COVID-19 cases and the Paediatric Department for pediatric COVID-19 cases. The rest of the hospitals offered the same services as usual, except that every patient being admitted was instructed to do a Polymerase Chain Reaction (PCR) test before admission. If the PCR was positive, the patient would be transferred to Obour Specialized Isolation Hospital.

Ain Shams University designated some of its hospitals and ICUs as “Isolation Hospitals” for the management of COVID-19 patients, which became the target for many, if not all, of the COVID-19 research studies. In view of the overwhelming number of research protocols, more than one research protocol targeted the same population in the same isolation hospital or ICU. In a few instances, the same PI was involved in more than one study. While there are no regulations against this, the REC was concerned about the involvement of the same patient in more than one study. The FMASU REC did not allow enrolment of subjects in more than one ongoing clinical trial. This decision conflicts with Cinnella and Gertner who reported that co-enrolment does not affect the safety of patients, the study outcome, or side effects, provided that the inclusion and exclusion criteria are appropriately set (10, 11).

Before COVID-19, the REC stressed on selecting the appropriate control groups for the study, especially regarding how healthy controls were selected. For the COVID-19 protocols the controls were usually sick patients, hence risk mitigation was the major issue. During review of COVID-19 protocols, the REC noticed that several RCTs control groups did not receive interventional medication and had similar characteristics and inclusion criteria, e.g., the severity of the disease. To decrease the number of subjects included in the studies, the REC suggested the use of the same set of controls, whenever applicable and possible, in more than one study. This meant that the subjects were enrolled only once, and their data was shared with other investigators performing their studies at the same time in the same place. The intervention arm, however, included different subjects, receiving different medications according to the trial they were enrolled in. The committee also required the intervention group to control group ratio to be 1:1, and not more, in order to avoid enrolment of more subjects than required in the RCTs.

In many clinical trials placebo control arm is recommended, especially where the effect of the drug is still not well-documented, or as obvious as in cancer research where a drug causes shrinkage of the tumor (12). For the placebo-controlled trials, the REC decided that all participants must receive the updated standard of care treatment as per the Egyptian MOHP protocol for COVID-19 patients, while the participants receive the new drug under trial as add-on therapy. This way the clinical trial design was an add-on design, where the controls received the standard care MOHP protocol, rather than receiving a sham medication. The REC thought that this could minimize the risk, although the standard protocols at the start of the pandemic had no clear evidence of benefit.

The inclusion of the Egyptian MOHP COVID-19 management protocol was a challenge to the investigators, as the treatment protocol was constantly updated according to new information arising in that arena. The FMASU REC recommended that the standard of care set by the Egyptian MOHP always be updated in the submitted protocols and provided to all COVID-19 study participants.

Regarding the clinical trial endpoints, the FMASU REC had lengthy discussions on the use of measurable achievement vs. patient-related outcomes in the pandemic research. Several protocols used “the time to clinical improvement” as the measure for outcome. Clinical improvement in some studies was defined as the time from randomization to either an improvement of two points on a six or eight-category ordinal scale. Some members of the committee considered the scale as very subjective and required the use of more objective or measurable outcomes. The FMASU REC resorted to the time to clinical improvement scale, in addition to the patient-related outcome scales, the investigators should use more objective outcome measures in their studies, such as persistent positive PCR tests after treatment, or time until the emergence of antibodies against the novel virus. The use of objective tests as outcomes would be a dynamic process as new information is published.

Ensuring the provision of a clear informed consent form was a big challenge for the committee, and certainly for the investigators as well. The motivational force behind the willingness of the patients to enroll in the clinical trials is complex, and therapeutic misconception had to be clearly avoided in the consent language. Due to the scarcity of available information about the virus and the use of novel drugs, the committee ensured that the investigators simplify the information provided to the participants in a manner that the participants could comprehend, as there were so many unknown facts about the virus. The committee understood how challenging it was to describe and explain unknown risks to the potential participants. Still, the FMASU REC required the explanation of risks to be clear and in a language the participants could understand. Furthermore, the prospect of direct benefits was in no way to be promised or overestimated. The FMASU REC members conducted a meticulous review of the wording of the informed consent to ensure the message was clearly communicated to the potential participants and their guardians, regarding the lack of scientific evidence of the efficacy of the used drugs, as well as the unknown side effects, while still providing convincing rationale for the performance of the study.

Additional minor concerns in some of the studies included the completion of a diary for drug doses to be completed by the patients. The FMASU REC was concerned about how a severely ill participant would record his/her daily doses of drugs under trial, especially for the non-educated participants. The FMASU REC requested that this be confined to moderate or highly educated cases only.

The FMASU REC requested the timely reporting of all adverse events as soon as they occurred, not only the serious ones as per the standard procedures which assign a medical monitor in all COVID-19 clinical trials. The medical monitor should be a medical doctor, not involved in the study, but who observes the progress of the study and provides reports to the FMASU REC in case of adverse events. In studies assessing psychological risks to healthcare workers, the FMASU REC requested that participants who tested positive on screening, receive free management and referral to receive psychiatric help if proven to be suffering from anxiety or depression.

Common modifications and clarifications requested by the FMASU REC were the inclusion criteria and the age range of the recruited subjects. Enrolment of subjects not receiving other medicines under trial was a challenge during review. Detailed data of the study procedures were requested in many submitted research studies. More frequent progress reports were requested.

To mitigate risks, more frequent electrocardiograms, complete blood counts, x-rays, and chest Computed Tomographies were requested to safeguard against serious adverse events of the drugs under investigation, such as Hydroxychloroquine and other repurposed drugs. Regarding the control groups in the RCTs, the REC recommended that controls obtain the standard MOHP protocol for COVID-19 management. The REC requested monthly progress reports and swift notification of serious adverse events.

The innovative approaches adopted by the REC in the earlier wave of the COVID-19 pandemic were acceleration of submission of pre-requisite paperwork needed for application, increased frequency of virtual meetings, expansion of meeting agendas, direct contact with the PIs by phone and fast-track review within 1-7 days (compared to the usual 1-month review of clinical trials according to the SOPs.

FMASU REC experience, being active for 13 years since 2007 would be beneficial for other Egyptian RECs, numbering approximately 85 committees in 2021 with variable experience. Seventy Egyptian RECS are linked through a non-governmental body named the Egyptian Network of Research Ethics Committees (also known as ENREC), established in 2008, that enhances REC networking, standardizing the SOPs among RECs all over Egypt, the exchange of knowledge of research review challenges and obstacles, as well as finding solutions through regular annual meetings (13). Thirty-five RECs are registered with the MOHP.

Data confidentiality is fundamental in both COVID-19 and non-COVID-19 research to protect the life, health, dignity, integrity, right to self-determination, privacy, and confidentiality of personal information of research subjects. It has been practiced since the establishment of the committee according to the Declaration of Helsinki (14).

During the review of COVID-19 research, the REC was more diligent in reviewing the parts of the protocol where the investigators detailed the precautions for data confidentiality, including databases and computer files, as well as paper copies of questionnaires and informed consents. REC members followed the REC review checklist to review the protocols with confidentiality adequately listed in the checklist. Digital tracking technologies were not allowed, so there were no concerns regarding generated data confidentiality.

Some of the strengths of this analysis at the organizational level were REC resilience and at the research level, the researchers continued conduct of research in spite of the discussed challenges in order to generate knowledge for this new disease and to accomplish investigator career progress. Regarding the research participants: COVID-19 investigation results, including PCR, lab and radiology and all medications given to research participants were provided free of charge. Participants were offered the autonomy to participate in research under strict REC oversight during that period, which was characterized by little and misinformation. Regarding inclusiveness and diversity, participants included healthcare professionals, literate and illiterate patients, and the elderly without discrimination in research enrolment while maintaining equity of healthcare.

Our research prioritization during the COVID-19 pandemic is in line with that of Kheng-Wei Yeoh and Ketan Shah, who provided recommendations for RECs on research prioritization and fast-tracking research, without compromising the participants' safety and well-being. Priority should be given to research that helps find a cure for the patients, while other research should be re-evaluated for public health concerns and precautions incorporated into the studies. As for the design, the authors recommend incorporating the study design into clinical care and look for new information due to the increased demand (15).

The Pan American Health Organization (PAHO), website on April 15, 2020 published guidance for the development of SOPs for RECs for the review of research during the COVID-19 pandemic. In addition to the revised SOPs, the PAHO recommended that RECs should accelerate reviews and initiate a system for follow up on COVID-19 research (16).

Due to confidentiality issues, the authors provided minimal details about the studies. We would have liked to expand on the types of research, but many of the studies included new drugs about which we could not provide details. Another limitation is that this article's scope is limited to the performance of the REC and the challenges it faced, rather than a predesigned research study or survey.

## Conclusion

During the COVID-19 pandemic the FMASU REC was overwhelmed with a huge number of COVID-19 -related research protocols. The increased amount of research protocols to be reviewed in a short time presented several logistic and ethical challenges. The committee had to adopt different methods of review to ensure adherence to the ethical principles. The ethics training background of the members proved beneficial to balance the risks and benefits to the patients among novel ethical dilemmas.

## Author Contributions

All authors listed have made a substantial, direct and intellectual contribution to the work, and approved it for publication.

## Funding

This study was self-funded by the authors, who have not received any monetary or financial support for the writing and publishing.

## Conflict of Interest

The authors declare that the research was conducted in the absence of any commercial or financial relationships that could be construed as a potential conflict of interest.

## Publisher's Note

All claims expressed in this article are solely those of the authors and do not necessarily represent those of their affiliated organizations, or those of the publisher, the editors and the reviewers. Any product that may be evaluated in this article, or claim that may be made by its manufacturer, is not guaranteed or endorsed by the publisher.

## Abbreviations

RECs: Research ethics committees
EUREC: European Network of Research Ethics Committees
FMASU REC, Faculty of Medicine: Ain Shams University Research Ethics Committee
ICU: Intensive care units
MOHP: Ministry of Health and Population
PAHO: Pan American Health Organization
PCR: Polymerase Chain Reaction
PI: Principal investigator
RCTs: Randomized controlled trials
SOPs: Standard operating procedures
WHO: World Health Organization.
